# Activation of Pyramidal Neurons in Mouse Medial Prefrontal Cortex Enhances Food-Seeking Behavior While Reducing Impulsivity in the Absence of an Effect on Food Intake

**DOI:** 10.3389/fnbeh.2016.00063

**Published:** 2016-03-30

**Authors:** Daniel M. Warthen, Philip S. Lambeth, Matteo Ottolini, Yingtang Shi, Bryan Scot Barker, Ronald P. Gaykema, Brandon A. Newmyer, Jonathan Joy-Gaba, Yu Ohmura, Edward Perez-Reyes, Ali D. Güler, Manoj K. Patel, Michael M. Scott

**Affiliations:** ^1^Department of Pharmacology, University of VirginiaCharlottesville, VA, USA; ^2^Department of Anesthesiology, University of VirginiaCharlottesville, VA, USA; ^3^Department of Neuropharmacology, Hokkaido University Graduate School of MedicineSapporo, Japan; ^4^Department of Biology, University of VirginiaCharlottesville, VA, USA

**Keywords:** prefrontal cortex, DREADD, food, operant behavior, impulsivity

## Abstract

The medial prefrontal cortex (mPFC) is involved in a wide range of executive cognitive functions, including reward evaluation, decision-making, memory extinction, mood, and task switching. Manipulation of the mPFC has been shown to alter food intake and food reward valuation, but whether exclusive stimulation of mPFC pyramidal neurons (PN), which form the principle output of the mPFC, is sufficient to mediate food rewarded instrumental behavior is unknown. We sought to determine the behavioral consequences of manipulating mPFC output by exciting PN in mouse mPFC during performance of a panel of behavioral assays, focusing on food reward. We found that increasing mPFC pyramidal cell output using designer receptors exclusively activated by designer drugs (DREADD) enhanced performance in instrumental food reward assays that assess food seeking behavior, while sparing effects on affect and food intake. Specifically, activation of mPFC PN enhanced operant responding for food reward, reinstatement of palatable food seeking, and suppression of impulsive responding for food reward. Conversely, activation of mPFC PN had no effect on unconditioned food intake, social interaction, or behavior in an open field. Furthermore, we found that behavioral outcome is influenced by the degree of mPFC activation, with a low drive sufficient to enhance operant responding and a higher drive required to alter impulsivity. Additionally, we provide data demonstrating that DREADD stimulation involves a nitric oxide (NO) synthase dependent pathway, similar to endogenous muscarinic M3 receptor stimulation, a finding that provides novel mechanistic insight into an increasingly widespread method of remote neuronal control.

## Introduction

The medial prefrontal cortex (rodent prelimbic and infralimbic cortex, mPFC) is implicated in the control of a host of executive cognitive functions including reward valuation (Grabenhorst and Rolls, 2011), decision-making (Euston et al., 2012), memory extinction (Maroun, 2013), attention (Clark and Noudoost, 2014), task switching (Kehagia et al., 2010), and the regulation of affect (Price and Drevets, 2012). Accordingly, alteration in prefrontal function has been noted in disorders associated with dysregulated executive control including addiction, post-traumatic stress disorder, mood disorders, and compulsive disorders. Likewise, manipulation of mPFC neurons pharmacologically, optogenetically, electrophysiologically, and by lesioning in model organisms has demonstrated involvement of this brain area in many aspects of executive function (Kesner and Churchwell, 2011; Cassaday et al., 2014; Riga et al., 2014).

In addition, the mPFC is involved in modulating food intake and food reward. Pharmacological and optogenetic manipulation of the mPFC can drive unconditioned feeding in sated animals (Mena et al., 2011, 2013; Land et al., 2014). Cued feeding has also been shown to require mPFC activity (Petrovich et al., 2007). Operant responding for palatable food is also regulated by the mPFC; stimulating μ-opioid receptors (MORs) in the ventral mPFC increases the breakpoint on a progressive ratio (PR) operant schedule (Mena et al., 2013), while antagonizing opioid signaling in the mPFC via naltrexone infusion decreases operant responding for a food reward (Blasio et al., 2014). Likewise, infusion of the dopamine type-1 receptor antagonist SCH-23390 attenuates the expression of instrumental learning (Baldwin et al., 2002). Furthermore, the activity of mPFC neurons tracks with reward receipt in rats, as the animal engages the mPFC in calculating the costs associated with reward seeking (Burgos-Robles et al., 2013; Horst and Laubach, 2013). Reinstatement of palatable food seeking, a model of dietary relapse, is associated with increased c-Fos induction in the mPFC (Cifani et al., 2012), and optogenetic manipulation of mPFC function interferes with reinstatement in a stimuli-specific manner (Calu et al., 2013). These data and others strongly implicate the mPFC, and especially metabotropic receptor signaling within the mPFC, in regulating food intake and reward. However, a mechanistic understanding of these results on a cellular/circuit level is complicated by the fact that within the mPFC both glutamatergic pyramidal projection neurons and GABAergic local interneurons express dopamine and opioid receptors, as well as other metabotropic receptors likely involved in modulating food intake and reward, prohibiting assignment of function to a particular cell type (Steketee, 2003).

We hypothesized that selectively depolarizing mPFC pyramidal neurons (PN) in the mouse would be sufficient to modulate performance in both feeding assays and in assays that measure how the mPFC regulates food seeking behavior. Using an adeno-associated virus (AAV) construct encoding a G_q_-coupled Designer Receptor Exclusively Activated by Designer Drugs (hM3D(Gq)-DREADD; Armbruster et al., 2007; Alexander et al., 2009; Krashes et al., 2011) under control of the calcium-calmodulin dependent protein kinase II alpha (CamKIIα) promoter, we investigated whether increasing the excitability of mPFC PN via metabotropic signaling could selectively alter behavior.

Our results demonstrate that DREADD-driven neuronal stimulation is sufficient to enhance both the effort devoted to food seeking and the reinstatement of cued food seeking behavior, while reducing motor impulsivity. Intriguingly, our stimulation paradigm spared effects on unconditioned food intake, as well as behaviors associated with anxiety, depression, and social interaction, which have been linked to mPFC function. Thus, these results suggest that Gq driven neuronal stimulation of CamKII PN affects food seeking behavior but not the ingestion of food.

## Materials and Methods

### Animals

For all behavioral experiments, 9 week old male C57BL/6J mice were purchased from Jackson Laboratory (Bar Harbor, ME, USA). For studies assessing colocalization of DREADD or eYFP with dopamine receptor 1 (Drd1), we used mice expressing Cre recombinase under control of the Drd1 promoter, which have been described previously (Heusner et al., 2008). To test the AAV-DIO-Sun2myc vector (below), we used Vgat-ires-Cre mice from Jackson Laboratory (Stock No. 016962) crossed with lox-tdTomato mice (Jackson Laboratory, Stock No. 007914). Mice were housed in the Jordan Hall vivarium at the University of Virginia on a 12:12 light:dark schedule with lights-on at 09:00 with free access to food and water, unless otherwise noted. All testing was performed during the light portion of the light:dark cycle. All experiments were performed in accordance with Association for Assessment of Laboratory Animal Care policies and approved by the University of Virginia Animal Care and Use Committee. Four separate cohorts of mice were used for behavioral and electrophysiological experiments. Cohort 1 participated in the following experiments, in the following order: Operant Conditioning, Binge-Like Assay (0.5 mg/kg CNO), Open Field (0.5 mg/kg CNO), Social Interaction (0.5 mg/kg CNO). Cohort 2 participated in the following experiments, in the following order: Reinstatement, Binge-Like Assay (2.5 mg/kg CNO), Fast-Refeed, Open Field (2.5 mg/kg CNO), Social Interaction (2.5 mg/kg CNO). Cohort 3 only participated in the Impulsivity Assay. Cohort 4 only participated in electrophysiological studies.

### Adeno-Associated Virus Vectors and Stereotaxic Viral Injection

For expression of hM3D(Gq) in mPFC PN for behavioral assessment, we used the AAV-CaMKIIa-HA-hM3D(Gq)-IRES-mCitrine vector (UNC Vector Core). For colocalization studies, we used the AAV-CaMKIIa-hM3D(Gq)-mCherry and AAV-CaMKIIa-EYFP vectors (UNC Vector Core). Likewise, for electrophysiological experiments we used the AAV-CaMKIIa-hM3D(Gq)-mCherry vector, as the mCherry fluorophore is more readily detectable by epifluorescence. Our Cre recombinase dependent vector AAV-DIO-SUN2-myc was also produced by the UNC Vector Core. Virus was delivered via stereotaxic injection. Mice were anesthetized with ketamine/dexmeditomidine solution and mounted on a stereotaxic apparatus. Anesthesia was maintained by inhaled isoflurane for the duration of the procedure. A small hole was opened in the skull, and a pulled glass micropipette was lowered to the target site (from Bregma, +2.05 mm anterior, ±0.35 mm lateral, and −1.50 mm ventral of dura). 200 nl of virus was injected with pressure at a rate of ~50 nl/min. After injection the micropipette was maintained in place for 4 min before retraction. This procedure was repeated bilaterally. The incision was then cleaned and closed with a surgical staple. Mice were given atimpamezole and ketoprofen immediately following surgery, and ketoprofen was administered for 3 days post-surgery. Mice were allowed to recover for at least 3 weeks following surgery before commencement of behavioral testing.

### Generation of AAV-DIO-Sun2myc

We developed a novel cre dependent viral marker for assessing the expression of the Drd1 receptor in the mPFC. A mouse Sun2 (Genbank AY682987)-C-terminal myc tagged fusion cDNA was synthesized by Genscript (Piscataway, NJ, USA), containing both 5′ NheI and 3′ AscI restriction sites. This DNA fragment was then cloned into the unique NheI-AscI restriction sites of pAAV-EF1a-double floxed-hChR2(H134R)-EYFP-WPRE-HGHpA (gift from Karl Deisseroth—Addgene plasmid #20298). The subsequent construct then contained the Sun2-myc fusion (removing hChR2(H134R)-EYFP) in inverse orientation from the EF1a promoter. Cre recombinase expression then reverses the orientation of the Sun2-myc cassette and allows expression. AAV viral particles were then prepared by the University of North Carolina’s Vector Core Facility.

### Single-cell RT-PCR

Individual mCherry-labeled neurons were harvested from mouse prelimbic cortex acutely, essentially as described previously (Wang et al., 2013; Kumar et al., 2015). Briefly, single fluorescent cells were identified in the recording chamber of a fluorescence microscope (Zeiss Axioimager FS) and aspirated into a glass capillary. The tip was broken and contents expelled into a sterile Eppendorf tube containing dNTPs, BSA, RNaseOUT, oligo-dT and random hexamers; this pre-RT mixture was incubated at 65°C for 5 min then cooled to 4°C for 1 min. First strand cDNA synthesis was performed with Superscript III Reverse Transcriptase, RNA was digested with RNase H and cDNA and stored at −20°C. Analysis of multiple genes in single cell assays employed multiplex nested single cell RT-PCR (sc-PCR). All primer sets were independently validated and no-template negative controls were included for each reaction. Primers for VGLUT1 and GAD65a were previously reported (Cabezas et al., 2013), as were primers for GAD65b, parvalbumin (PARV), somatostatin (SOM), and VIP (Sosulina et al., 2010), and for GAPDH, VGLUT2, and GAD67 (Wang et al., 2013; Kumar et al., 2015).

### Electrophysiology

Coronal brain slices (300 μm) containing the mPFC were prepared from AAV hM3Dq-DREADD-injected mice. Animals were euthanized with isoflurane, decapitated, and their brains were rapidly removed and submerged in ice cold artificial cerebral spinal fluid (ACSF) containing (in mM): 125 NaCl, 2.5 KCl, 1.25 NaH_2_PO_4_, 2 CaCl_2_, 1 MgCl_2_, 0.5 L-ascorbic acid, 2 pyruvate, 10 glucose, and 25 NaHCO_3_ (oxygenated with 95% O_2_ and 5% CO_2_). Slices were prepared using a Vibratome (Vibratome 1000 Plus), transferred to a chamber containing oxygenated ACSF, incubated at 37°C for 35 min, and then stored at room temperature. For recordings, slices were held in a small chamber superfused with heated (32°C) oxygenated ACSF at 3 mL/min. For electrophysiology experiments, mPFC neurons were visually identified using fluorescent microscopy using a Zeiss Axioscope microscope (Zeiss, Oberkochen, Germany). Whole-cell current clamp recordings were performed using an Axopatch 700B amplifier (Molecular Devices) using pCLAMP 10 software (Molecular Devices) and a Digidata 1322A (Molecular Devices). Electrodes were fabricated from borosilicate glass using a Brown-Flaming puller (model P97, Sutter Instruments Co). Electrodes (3.0–3.5 MΩ) were filled with (in mM): 120 Kgluconate, 10 NaCl, 2 MgCl_2_, 0.5 K_2_EGTA, 10 HEPES, 4 Na_2_ATP, 0.3 NaGTP (pH adjusted to 7.2 with KOH). APs were evoked using a current injection step to 300 and action potential parameters measured as previously described (Hargus et al., 2013).

### Clozapine-N-Oxide (CNO)

CNO was purchased from Sigma (Cat. No. C0832–5MG) and dissolved in saline at a concentration of 1 mg/ml, then diluted to either 0.1 mg/ml (for 0.5 mg/kg injections) or 0.5 mg/ml (for 2.5 mg/kg injections).

### Free Feeding Assays

All behavioral testing for the binge-like and fast-refeed assays occurred in the home cage, in the home room.

### Binge-like Feeding Assay

The Binge-like Assay was performed as previously described (Gaykema et al., 2014). On the night before testing, mice (*N* = 10) received a small (<0.1 g) sample of either a highly palatable high fat, high sugar diet (HFD; Research Diets Inc., D12331, 5.56 kcal/g) in a petri dish to familiarize them with this novel food. At lights-on all food was removed, and mice received an injection i.p. of either saline or CNO, equal volume. After 30 min mice were challenged with ~1.5 g of pre-weighed HFD in a petri dish, and allowed to consume. After 15 min, 30 min, and 1 h the food was removed and weighed, then replaced in the cage. After 1 h mice were returned to ad lib chow feeding. The experiment was repeated for 2 days total, such that each mouse received HFD + saline and HFD + CNO. Mice were assigned randomly to begin the experiment on either saline or CNO.

### Fast-Refeed Assay

On the day before testing, food was removed at 15:30 (5.5 h before lights off). At lights-on on the following day, mice (*N* = 10) received an injection i.p. of either saline or CNO. After 30 min mice received ~3 g of pre-weighed chow in a petri dish, and were allowed to consume. After 15 min, 30 min, and 1 h the food was removed and weighed, and mice were returned to ad lib chow feeding. The experiment was repeated for 2 days total, such that each mouse received chow + saline and chow + CNO. At least 2 days of ad lib feeding separated each testing day. Mice were assigned randomly to begin the experiment on either saline or CNO. The Fast-Refeed Assay was performed 3 days after the Binge-Like Assay.

### Operant Conditioning

Behavioral testing for the operant conditioning assay occurred in a dedicated behavior room, separate from the home room. Testing was performed in sound attenuated boxes (Med-Associates, St. Alban, VT, USA). Each box was equipped with three nosepoke holes arranged in a line on one side of the chamber, and a food magazine on the opposite side. Each nosepoke hole, as well as the magazine, was equipped with an infrared beam break detector. Three days prior to training, mice were placed on food restriction, with access to regular chow for 3 h/day. Mice were trained for 1 h/day, every day. Prior to any training, mice initially underwent extinction training in order to extinguish any innate preferences for any of the nosepoke holes. In this phase, a nosepoke did not result in any reward delivery. After passing extinction (fewer than 10 pokes in any hole in a given session), mice were passed on to fixed ratio (FR) training. FR training proceeded in three stages, FR1, FR3, and FR5. In FR1, a single nosepoke in the center hole resulted in delivery of a food reward (Bio-Serv, Cat. No. F05301) to the magazine. In FR3, three nosepokes are required for a reward, and in FR5 five nosepokes are required. A mouse was considered to have passed a stage when it attained 30 rewards in a single session. Mice were moved from FR1 to FR3 after passing FR1 one time, moved from FR3 to FR5 after passing FR3 twice, and moved from FR5 to PR testing after passing FR5 three times. In PR testing, the number of nosepokes required for a reward increased progressively during a session, on the following schedule: 5 pokes for the first reward, then 10, 20, 30, 50, 70, 100, 130, 170, 210, 260, 310, and finally 370. PR testing lasted for a maximum of 2 h, and could terminate early if the mouse did not complete any given stage in 30 min or less. Mice underwent 2 days of PR testing on food restriction alternating CNO/saline treatment, with half receiving CNO on the first day and half receiving saline, followed by 2 days of PR testing after return to ad lib feeding, also alternating CNO and saline. The chambers were cleaned between each mouse with Minncare disinfectant to remove residual odors.

### Reinstatement

The Reinstatement Assay is based on a previously reported assay (Martín-García et al., 2011), with some alterations. The primary difference is that our mice were maintained on ad lib feeding throughout the assay, rather than beginning the initial training period on food restriction. The reinstatement experiment consisted of three stages: Acquisition, Extinction, and Reinstatement. During Acquisition, the following program was used: a nosepoke in the center hole resulted in delivery of a reward pellet and simultaneous illumination of the nosepoke hole. Illumination was maintained for 2 s. Pokes into the left and right holes were recorded, but did not result in any action. After reward delivery, the mouse was required to enter the food reward hopper before the program would continue. After hopper entry, a 10 s hold period followed, in which nosepokes were counted but did not result in any action. After the 10 s hold, the cycle began anew, and nosepokes could again elicit reward delivery and illumination. Acquisition was considered successful when pokes in the active, center hole were 70% or greater than total nosepokes (including pokes in the left and right holes), and total rewards obtained in a session remained within 20% of the 3 day running average. After Acquisition, mice passed to Extinction, in which nosepokes were counted, but did not result in reward delivery or nosepoke illumination. Extinction continued until total pokes in the center hole were less than 30% of total pokes on the center hole during completion of Acquisition for 3 consecutive days. After Extinction, mice underwent a single day of Reinstatement testing. Thirty minutes prior to testing, mice received either CNO or saline i.p. Mice were assigned to the CNO or saline group prior to testing in order to achieve a comparable distribution of responding during Acquisition in both groups. During Reinstatement, the center nosepoke hole was continuously illuminated and mice were allowed to freely respond for 1 h. No reward was delivered. Poking in all holes was recorded. The chambers were cleaned between each mouse with Minncare disinfectant to remove residual odors.

### 3-Choice Serial Reaction Time Task (Impulsivity Assay)

The Impulsivity assay was performed as previously described, with modifications as noted (Ohmura et al., 2009). Briefly, training is split into 13 stages. In stage 1, all nosepoke holes are illuminated, and a nosepoke in any hole results in reward delivery. In stage 2, only the center nosepoke hole is illuminated, and only pokes in this hole results in reward delivery. As training progresses, the duration of nosepoke illumination is progressively reduced, until in stage 13 it is illuminated for 0.5 s. Mice must refrain from poking until the hole is illuminated. After stage 2, the mouse must wait (the intertrial interval (ITI) being 5 s) to identify the correctly lit nosepoke hole prior to poking, as one of the 3 holes is then illuminated in pseudo random order. A premature poke results in a 5 s timeout and no reward. Likewise, mice must poke within 2 s of light illumination. No response also results in a timeout and no reward. During testing (following completion of stage 13) the ITI is lengthened to 7 s to produce a slight elevation in impulsive responding, allowing us to measure either an increase or a decrease in this behavior. Premature nosepoking is recorded during training, but data and comparisons reported here are only during the Testing phase, following completion of stage 13 and achievement of steady state responding. Mice receive either saline, 0.5 mg/kg CNO, or 2.5 mg/kg CNO i.p. 30 min before testing. Mice alternate between saline and CNO, again balancing initial treatment. The chambers were cleaned between each mouse with Minncare disinfectant to remove residual odors. In this study, we used a performance threshold of <1.75 s mean correct response latency, >70% percent accuracy for inclusion in testing. We did not apply a threshold for omission errors.

### Open Field

For the Open Field assay the lights in the room were turned down and mice were allowed to acclimate for at least 2 h prior to testing. The Open Field chamber was constructed as previously described (Golden et al., 2011). Thirty minutes after injection of either CNO or saline, mice were placed into the open field chamber, next to the edge, and allowed to explore for 5 min while movement was recorded using EthoVision XT tracking Software (Noldus, Leesburg, VA, USA). The open field chamber was cleaned between each mouse with Minncare disinfectant to remove residual odors. During testing, the lights in the room were turned down, providing a dim light environment.

### Social Interaction

The Social Interaction task was performed in our open field chamber, as previously described (Golden et al., 2011). Prior to the social interaction test, all mice were brought to the behavior room and allowed to acclimate for at least 1 h. Mice received an injection i.p. of either saline or CNO 30 min prior to starting the test. In brief, the chamber was prepared with an empty restrainer in the interaction zone (IZ), against the wall. A DREADD receptor injected mouse was place in the chamber, adjacent to the wall opposite the restrainer (as in the open field assay), and allowed to explore for 150 s. The DREADD mouse was then removed to the home cage for 30 s, while the empty restrainer was replaced with a new, clean restrainer. A novel mouse (129/SJL) was placed in the new restrainer, and the DREADD mouse was returned to the chamber and allowed to explore for 150 s. Behavior and motion were recorded using a video tracking system and Ethovision Software. The chamber was cleaned between each mouse with Minncare disinfectant to remove residual odors.

### Immunohistochemistry

Immunohistochemistry of mCitrine for analysis of DREADD expression was performed following standard procedures, as previously described (Gaykema et al., 2014). The following antibody was used for immunodetection: mCitrine (Living Colors anti-GFP, Clontech, #632592, 1:1000). Briefly, mice were perfused intracardially and brains were removed and sectioned on a vibratome. Forty micrometers (40 μm) thick sections were incubated in PBS containing H_2_O_2_ and sodium azide to suppress endogenous peroxidase activity. Sections were then incubated in blocking buffer containing 1% normal goat serum and 0.5% Triton X-100 overnight at 4°C. Sections were washed in PBS, and incubated in primary antibody (see above) overnight at 4°C. Sections were washed again and incubated overnight at 4°C in goat anti-rabbit biotinylated secondary antibody (1:500), then developed using Ni-DAB staining and the Vectastain Elite ABC staining kit from Vector Labs, according to manufacturer’s instructions (Cat. No. PK-6100, Burlingame, CA, USA). Immunohistochemistry of SUN2-myc and mCherry for analysis of colocalization of DREADD and DR1 was performed similarly, with the following modifications: (1) Sections were initially treated in NaBH_4_, diluted to 1 mg/ml in PBS, for 10 min to reduce autofluorescence, (2) Incubation in H_2_O_2_ was omitted, as was development in Ni-DAB, and (3) The following antibody combinations were used: for eYFP/SUN2-myc tissue, rabbit anti-myc (1:1000; Abcam; catalog no. ab9106) plus Cy3-conjugated goat anti-rabbit antibody (1:1000; Jackson ImmunoResearch; catalog no. 111–165–144) was used to visualize AAV-DIO-SUN2-myc infected neurons, while the epifluoresence of eYFP was used to visualize AAV-CaMKIIa-EYFP infected neurons. For DREADD/SUN2-myc tissue, rabbit anti-dsRed (1:5000; Clontech; catalog no. 632496) plus Cy3-conjugated goat anti-rabbit (1:1000; Jackson ImmunoResearch; catalog no. 111–165–144) was used to amplify mCherry fluorescence and thereby visualize AAV-CaMKIIa-hM3D(Gq)-mCherry infected neurons, followed by Alexa Fluor 488-conjugated mouse anti-myc (1:1000; Cell Signaling; catalog no. 9b11) to visualize AAV-DIO-SUN2-myc infected neurons. Sections were imaged on either an Olympus BX51 microscope (for Ni-DAB) or a Nikon Eclipse 80i microscope (for fluorescence).

### Statistical Analysis

Statistical analysis was performed with GraphPad Prism 6. In the Operant and Reinstatement assays, a two-way repeated measures analysis of variance (rmANOVA) was used to assess significance. In the Open Field and Social Interaction assays an unpaired *t*-test was used. In the Binge-Like and Fast-Refeed assays, which evaluated consumption at multiple time points under two conditions, a rmANOVA was used. In the Impulsivity assay, which measured behavior under several conditions (Saline, CNO 0.5 mg/kg, and CNO 2.5 mg/kg), an ANOVA was used to assess effects of treatment, with a Tukey’s *post hoc* test used to assess differences between treatments.

## Results

### Expression and Activity of hM3D_q_

**Figure 1:**
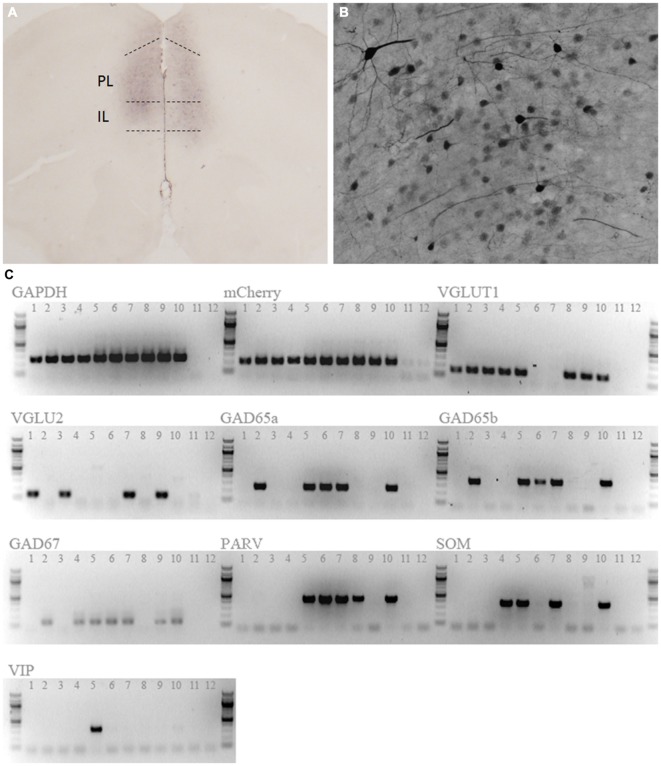
**Expression of hM3Dq designer receptors exclusively activated by designer drugs (DREADD).** Anti-mCitrine staining shows expression of the adeno-associated virus (AAV)-CaMKIIa-HA-hM3D(Gq)-IRES-mCitrine vector reporter in the mPFC **(A)**. Positively stained neurons had a characteristic pyramidal morphology **(B). (C)** Single cell RT-PCR of 10 mCherry-positive cells confirmed that DREADD-expressing cells were predominantly glutamatergic (9/10 cells). 8/10 cells also expressed additional tested markers. Lanes 1–10 are picked cells, while lanes 11 and 12 are no-template control reactions. GAPDH, Glyceraldehyde 3-Phosphate Dehydrogenase; VGLUT1, Vesicular Glutamate Transporter 1; VGLU2, Vesicular Glutamate Transporter 2;GAD65a, GAD65b, Glutamic Acid Decarboxylase 65; GAD67, Glutamic Acid Decarboxylase 67; PARV, Parvalbumin; SOM, Somatostatin; VIP, Vasoactive Intestinal Peptide.

We first confirmed expression and activity of the hM3Dq DREADD receptor. Immunohistological staining for the mCitrine reporter co-expressed with the hM3Dq-DREADD revealed positive staining bilaterally throughout the mPFC (Figure 1A). Positively stained cells had characteristic pyramidal neuron morphology (Figure 1B). Staining was observed predominantly in the prelimbic cortex, but included adjacent portions of the anterior cingulate and infralimbic cortex as well. Activity of the CamKIIα promoter is generally regarded to be restricted to excitatory neurons within the forebrain (Jones et al., 1994), and this promoter is frequently used to target AAV vectors to PN within the PFC (for example, Van den Oever et al., 2013). Nonetheless, we sought to further confirm the phenotype of DREADD-expressing cells by single cell RT-PCR (scRT-PCR). mCherry positive cells were predominantly glutamatergic, with 9 of 10 sampled cells expressing either vesicular glutamate transporter 1 (VGLUT1) or VGLUT2, or both (Figure 1C), confirming that this vector is highly selective for glutamatergic neurons. Interestingly, a portion of the cells in this sample also expressed glutamic acid decarboxylase 65 (GAD65) and GAD67, as well as parvalbumin (PARV), somatostatin (SOM), and vasoactive intestinal peptide (VIP; Figure 1C), suggesting a perhaps unappreciated molecular heterogeneity within the pyramidal neuron population of the mPFC (see “Discussion” section).

**Figure 2:**
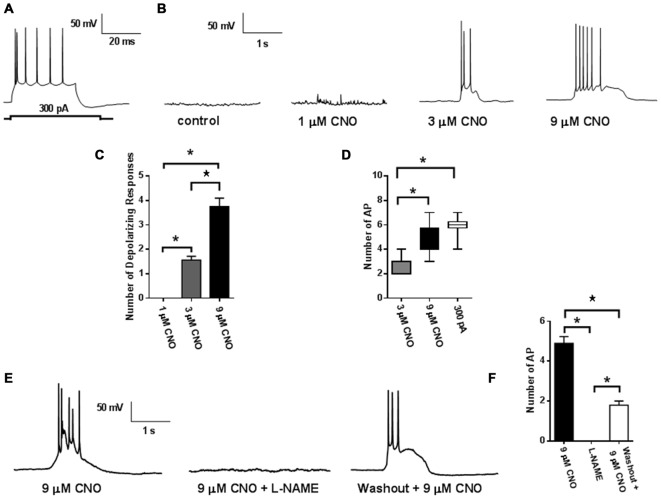
**Activity of hM3Dq DREADD.** Injection of a depolarizing current step (300 pA) evoked action potential firing in labeled mPFC neuron (*n* = 10; 6 mice: **A**). Application of CNO (5 mins) caused membrane depolarization and triggered action potential bursting (*n* = 19; 6 mice: **B**). **(C)** Quantification of the dose dependent increases in membrane depolarizations and firing rates **(D)**. CNO induced depolarizations and firing bursts were abolished by L-NAME (500 μM; **E**). **(F)** Quantification of reduction in action potential frequency by L-NAME. Values represent means ± SEM. *p < 0.001 ANOVA followed by Tukey’s *post hoc* test.

To confirm that application of CNO would evoke neuronal firing, membrane properties of labeled neurons were recorded under whole cell current clamp conditions (Figure 2). Injection of a depolarizing current step evoked firing in labeled neurons (Figure 2A; *n* = 10). Bath application of CNO (3 and 9 μM; *n* = 19) dose dependently evoked depolarizing events that triggered a dose dependent increase in bursts of action potentials (Figures 2B–D). Membrane properties and frequency of action potentials evoked using either a depolarizing current injection step or by CNO (9 μM) application were not different (Figure 2B, Table 1). We then investigated the mechanism of DREADD driven neuronal activation, to determine whether it resembled that of the endogenous M3 muscarinic receptor. Interestingly, similar to what has been observed following M3 receptor activation (Fassini et al., 2015), the effects of CNO (9 μM) were completely abolished by the nitric oxide (NO) inhibitor L-NAME and were partially reversible on washout (500 μM; *n* = 13: Figures 2E,F). These findings confirm that mPFC PN expressing DREADD can be activated in a dose dependent manner by CNO application, and that NO synthesis is required for the ability of the DREADD receptor to produce an increase in neuronal activity as has been recently suggested (Fassini et al., 2015).

**Table 1:** **Comparison of hM3D(Gq) activation to 300 pA current injection**.

	RMP	Amplitude (mV)	Threshold (mV)	Upstroke velocity (mV/ms)	Width (ms)	*N*
300 pA Current injection	−60.5 ± 0.1	49.1 ± 0.9	−39.0 ± 0.9	155.4 ± 3.6	2.3 ± 0.1	10
CNO 9 μM	−61.1 ± 0.2	48.8 ± 0.7	−38.8 ± 1.0	148.3 ± 4.2	2.3 ± 0.1	19

### Activation of mPFC PN Enhances Operant Performance for Food Reward

**Figure 3:**
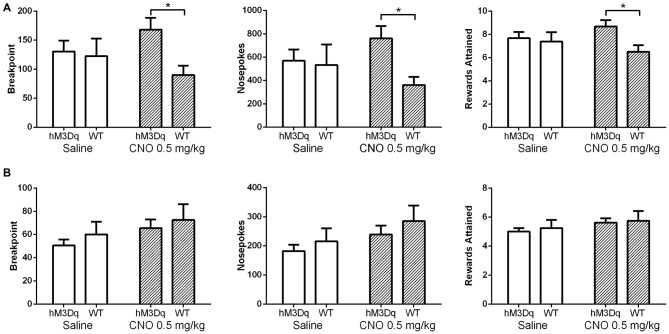
**Excitation of mPFC pyramidal neurons (PN) enhances operant responding.** Saline (white bars) had no effect on operant responding in DREADD-expressing mice (*N* = 16) relative to wildtype mice (*N* = 8) in either the food restricted **(A)** or fed **(B)** state, whether measured as breakpoint, total nosepokes delivered, or rewards obtained. In contrast, CNO (striped bars) did enhance operant responding in DREADD-expressing mice relative to wildtype mice in the food-restricted state, but not in the fed state. Error bars are ±SEM. *p < 0.05, 2-way rmANOVA followed by Sidak’s multiple comparisons test.

In our initial experiments, we wanted to test whether increasing mPFC pyramidal neuron activation would enhance conditioned food seeking; as prior work suggests the mPFC plays an important role in driving PR responding for reward. AAV hM3Dq-DREADD-injected mice (*N* = 16) were first trained in the absence of CNO stimulation on a FR schedule. Upon PR testing, CNO significantly enhanced operant responding for a highly palatable food reward in food restricted, DREADD-expressing mice relative to control, non-DREADD-expressing mice over the first two days of testing (Figure 3A). Statistical analysis revealed a significant interaction between DREADD expression and treatment, whether the data were evaluated as breakpoint (*F*_(1,22)_, *p* = 0.0133), total nosepokes (*F*_(1,22)_, *p* = 0.0197), or total rewards obtained (*F*_(1,22)_, *p* = 0.0178). This effect was driven by a significant difference in responding following CNO challenge between DREADD-expressing and wildtype mice (*p* < 0.05 for Breakpoint, Nosepokes, and Rewards; Sidak’s multiple comparisons test). Operant responding was not different between the DREADD-expressing and wildtype groups on saline, indicating that viral infection and DREADD expression alone do not impact operant behavior (*p* > 0.05 for Breakpoint, Nosepokes, and Rewards; Sidak’s multiple comparisons test). Furthermore, there was no difference in nosepokes into the inactive ports between the two treatments at any stage of PR testing (Table 2), demonstrating that the enhanced nosepoking was specifically directed at obtaining reward, rather than being a non-specific enhancement of nosepoking. The enhancing effect of CNO in DREADD-expressing mice starkly contrasts with results observed in control mice that did not express DREADD (*N* = 8). In contrast to the effects observed in food-restricted mice, CNO did not enhance operant responding in DREADD-expressing mice following return to ad lib feeding, as revealed by the absence of a significant interaction between DREADD expression and treatment, whether analyzed as breakpoint (*F*_(1,22)_, *p* = 0.8311), total nosepokes (*F*_(1,22)_, *p* = 0.7891), or total rewards obtained (*F*_(1,22)_, *p* = 0.7921; Figure 3B). As in the food-restricted state, CNO in the fed state had no effect on inactive port entries (Table 3). Taken together, these results demonstrate that enhancing pyramidal cell excitability in the mPFC via CNO-DREADD is sufficient to enhance operant responding in the food-restricted state, but not under ad-lib fed conditions. Furthermore, our results in control mice show that CNO alone has no significant effect on operant behavior in the absence of DREADD, in contrast to the related compound clozapine (den Boon et al., 2012).

**Table 2:** **Nosepokes into inactive ports during progressive ratio (PR) testing on food restriction**.

	Saline			CNO 0.5 mg/kg		
	*N*	Pokes	σ	*N*	Pokes	σ
FR5	16	2	1.51	16	2.88	4.94
FR10	16	1.56	1.63	16	2.63	2.78
FR20	16	3.69	4.32	16	5.13	5.02
FR30	16	5.06	4.57	15	6	5.82
FR50	15	11.8	7.41	15	9.6	6.88
FR70	14	14.5	10.1	15	12.33	6.53
FR100	12	23.08	20.5	15	26.87	32.8
FR130	8	17.38	11.7	13	28.46	29
FR170	5	28.6	20	8	34.88	22.3
FR210	2	15.5	20.5	5	22.6	5.32
FR260	2	19	22.6	3	21.67	13.3
FR310	1	13	N/A	1	7	N/A

**Table 3:** **Nosepokes into inactive ports during progressive ratio (PR) testing on ad lib feeding**.

	Saline			CNO 0.5 mg/kg		
	*N*	Pokes	σ	*N*	Pokes	σ
FR5	16	3.5	3.2	16	2.5	2.76
FR10	16	1.88	2.42	16	1.31	1.3
FR20	16	2.69	4.33	16	3.25	2.57
FR30	16	4.31	4.81	16	4.56	4.95
FR50	10	11.6	12	13	6.54	4.52
FR70	5	13.8	13.7	8	9.5	7.01
FR100	1	5	N/A	4	12.75	8.46
FR130	0	N/A	N/A	1	5	N/A

### Activation of mPFC PN Enhances Reinstatement of Palatable Food Seeking

**Figure 4:**
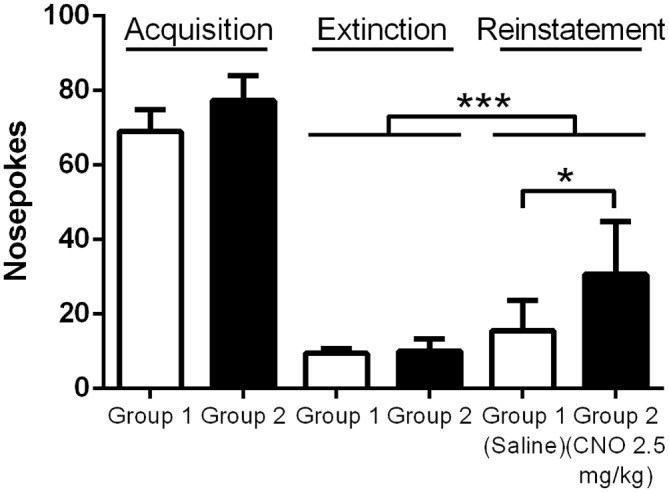
**Exciting mPFC PN enhances reinstatement of food seeking.** Note: Saline or CNO was administered only during the reinstatement test; the groups are separated during Acquisition and Extinction to demonstrate nose poking in each group. Mice in both the Saline (*N* = 8) and CNO (*N* = 9) groups successfully reinstated nose poking behavior during the Reinstatement test. Mice given CNO poked significantly more than mice given saline during the test. Error bars are ± SEM. *p < 0.05, ***p < 0.001, 2-way rmANOVA followed by Sidak’s multiple comparisons test.

We next wanted to test whether enhancing pyramidal neuron activation alters reinstatement responding for food reward. As prior work has demonstrated that the mPFC acts to drive reinstatement of drug seeking, we anticipated the same effect would be seen when animals are tested with palatable food. All mice (*N* = 17) successfully passed the reinstatement acquisition paradigm, with at least 70% of total nosepokes being made in the center, active hole. Average nose poking during the final three days of acquisition was significantly higher than average nose poking during the final three days of extinction, when no light cue was provided and no reward delivered (Figure 4; 73.4 ± 4.6 pokes vs. 9.7 ± 0.8 pokes), indicating the successful extinction of nose poking in the absence of a cue in all groups of animals. During reinstatement testing, when the light cue was continuously on for the duration of the test, mice in both the CNO and saline groups nose poked significantly more during the test than they did during the final day of extinction, indicating that the light cue was sufficient to reinstate food seeking behavior (*F*_(1,15)_, *p* = 0.0004; Figure 4). Importantly, CNO significantly enhanced nose poking relative to saline during testing, revealed by a significant interaction between time (Extinction vs. Test) and group (Group 1/Saline vs. Group 2/CNO; *F*_(1, 15)_, *p* = 0.0265; Sidak’s multiple comparisons test, *p* < 0.01; Figure 4). (CNO: 30.7 ± 4.7 pokes; Saline: 15.5 ± 2.9 pokes). Our results demonstrate that enhancing the excitability of mPFC PN via metabotropic signaling is sufficient to enhance reinstatement of food seeking behavior.

### Activation of mPFC PN Reduces Impulsive Responding for Food Reward

**Figure 5:**
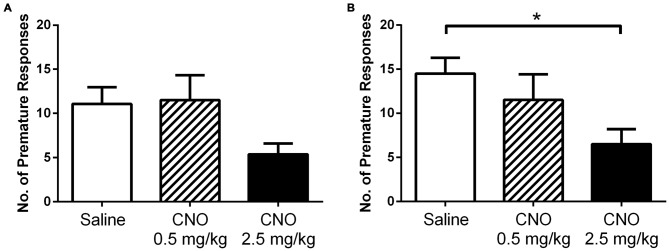
**Exciting mPFC PN reduces impulsivity.** CNO did not significantly alter premature responding in DREADD-expressing mice as a group (*N* = 12) in a 3-choice serial reaction time task when administered at 0.5 mg/kg (striped bars) or 2.5 mg/kg (black bars), relative to saline (white bars) **(A)**. However, in highly impulsive mice (*N* = 8) CNO did decrease premature responding **(B)**, an effect attributed to higher doses of CNO (2.5 mg/kg). Error bars are ± SEM. *p < 0.05 ANOVA followed by Tukey’s *post hoc* test.

Motor impulsivity is a behavioral characteristic modified by prefrontal cortex activity. Consequently, we hypothesized that similar to the observed effect on PR responding for palatable food, activation of mPFC PN would lead to a change in attention and impulsivity as well. We therefore trained DREADD-expressing mice in the 3-choice serial reaction time task, which measures a range of parameters including impulsivity, and then assessed behavior under control and stimulated conditions (Tsutsui-Kimura et al., 2009). We tested mice after vehicle saline and CNO at both a low (0.5 mg/kg) and high (2.5 mg/kg) dose. When we analyzed all mice for effects of treatment, we found no significant difference in premature responding (Figure 5A; *F*_(2,31)_ = 3.027; *p* = 0.0630), perseverative responses (*F*_(2,31)_ = 1.086; *p* = 0.3500), percent omissions (*F*_(2,31)_ = 0.2097; *p* = 0.8120), or total number of trials completed (*F*_(2,31)_ = 0.1973; *p* = 0.8220). As we observed a trend toward a reduction in premature responding in all mice, we reasoned that CNO may have a more dramatic effect on impulsivity in mice that tend to exhibit more premature responding in control (saline) conditions; in other words, highly impulsive mice. Therefore, we also analyzed for effects of treatment in only those mice with high rates of premature responding after saline treatment (e.g., those with premature responses totaling greater than one standard deviation above the mean of the low-impulsivity group following saline challenge [>6.5 premature responses, *N* = 8]). In this subgroup, we did indeed observe an effect of treatment on impulsivity (Figure 5B; *F*_(2, 19)_ = 4.122; *p* = 0.0362), which was driven by a decrease in premature responses when mice were treated with a high dose of CNO (*p* < 0.05). No significant effect of treatment was observed in this subgroup on perseverative responses (*F*_(2,19)_ = 1.450; *p* = 0.2594), percent omissions (*F*_(2,19)_ = 0.2525; *p* = 0.7794), or total number of trials completed (*F*_(2,19)_ = 0.3619; *p* = 0.7010). We therefore conclude that enhancing the excitability of mPFC PN decreases premature responding when required to obtain a food reward, particularly in mice with a predisposition toward impulsive responding. Furthermore, this effect is only apparent at higher doses of CNO that likely produce a greater degree of neuronal activation. Importantly, in contrast to the operant and reinstatement assays wherein CNO increased nosepoking, in the impulsivity assay CNO acted to decrease nosepoking. This demonstrates that CNO did not indiscriminately increase nosepoking. Rather, nosepoking behavior was modified according to the demands of the task.

While we did not test the effect of CNO in wildtype animals, the closely related compound clozapine has been shown to have no effect on premature responding in the 5-choice serial reaction time task, which is the basis of the 3-CSRTT (Baviera et al., 2008). Furthermore, clozapine increases omission errors in rats in an operant sustained attention task (Martinez and Sarter, 2008), which we also did not observe in the 3-CSRTT with CNO. This again supports the interpretation that the effects we have observed are not driven by CNO acting upon a pathway normally engaged by clozapine.

### Activation of mPFC PN Does Not Increase Unconditioned Hedonic or Homeostatic Food Intake

**Figure 6:**
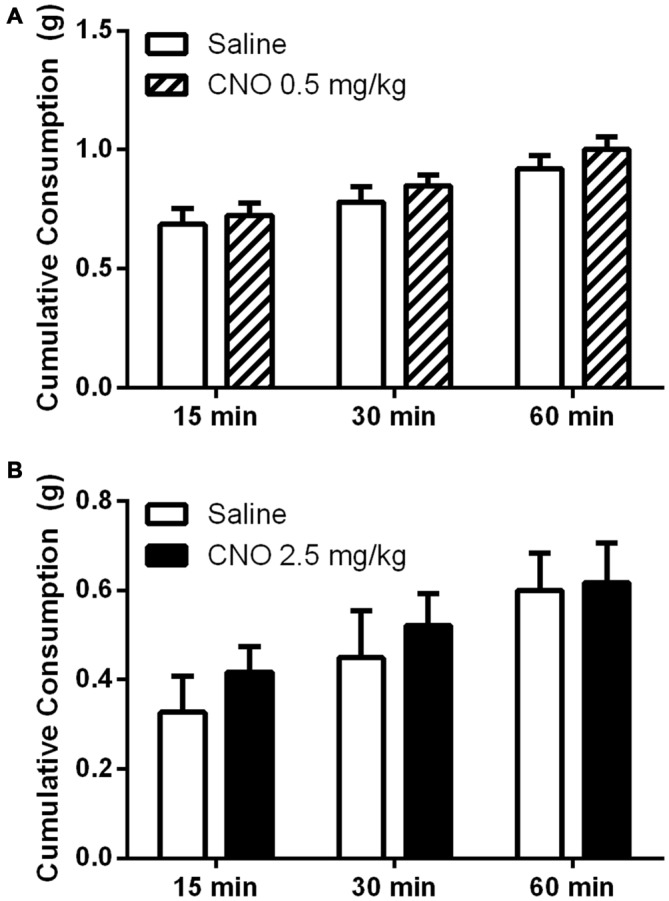
**Exciting mPFC PN does not alter binge-like consumption.** CNO did not alter consumption of a high fat diet in sated mice (*N* = 10) whether administered at 0.5 mg/kg **(A)** or 2.5 mg/kg **(B)** when food intake was measured after 15, 30, and 60 min. Error bars are ± SEM, rmANOVA.

**Figure 7:**
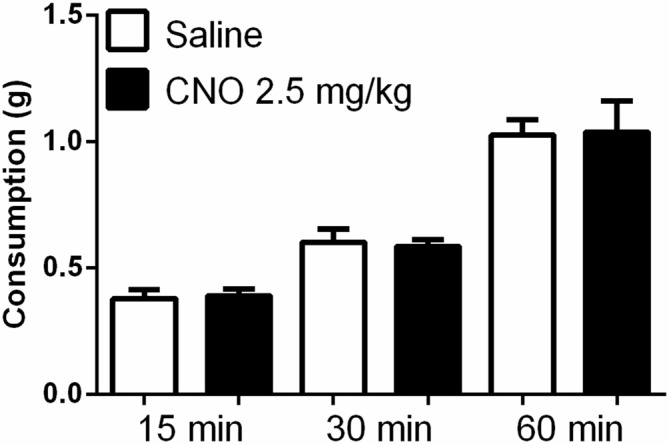
**Exciting mPFC PN does not alter food intake after fasting.** Regular chow intake in fasted mice (*N* = 10) was similar after saline (white bars) and CNO 2.5 mg/kg (black bars). Error bars are ± SEM, rmANOVA.

While prior studies have shown that pharmacological and optogenetic manipulation of the mPFC can alter free feeding, we wanted to investigate whether an increase in pyramidal neuron activation via metabotropic signaling would produce a similar effect. This approach differs from optogenetic stimulation, where neurons are stimulated to coordinately fire action potentials at specific frequencies, and from the application of non-cell selective receptor agonists and antagonists to the prefrontal cortex. Interestingly, unlike observations in prior work using optogenetic and pharmacological methods, we observed no effect on unconditioned food intake after administration of CNO, either in the fasted or fed state. In the fed state, CNO did not significantly alter intake of a highly palatable diet whether administered at 0.5 mg/kg (Figure 6A) or 2.5 mg/kg (Figure 6B), relative to saline. In an experiment examining the effects of a low dose of CNO (0.5 mg/kg), all mice consumed significant amounts of HFD over time, as revealed by a significant effect of time on food intake (*F*_(2,36)_ = 67.35; *p* < 0.0001); however, CNO did not modulate intake over the course of the hour, as shown by the lack of an effect of treatment (*F*_(1, 18)_ = 0.6698; *p* = 0.4238). In a separate experiment examining the effects of a higher dose of CNO (2.5 mg/kg), all mice again increased total consumption over the course of the experiment, as revealed by a main effect of time (*F*_(2,36)_ = 46.24; *p* < 0.0001), yet there was no effect of treatment on consumption (*F*_(1,18)_ = 0.2828; *p* = 0.6014). Likewise, in the fasted state mice ate significantly more chow over the course of the experiment, as shown by a significant main effect of time on food intake (*F*_(2,36)_ = 74.47; *p* < 0.0001); however, CNO (2.5 mg/kg) did not alter regular chow intake relative to saline, as demonstrated by the lack of a significant main effect of treatment on consumption (Figure 7, (*F*_(2,36)_ = 0.0016; *p* = 0.9677). These observations indicate that the activation of mPFC PN induced by our treatment is not sufficient to change unconditioned food intake.

### Activation of mPFC PN Does Not Alter Anxiety-Like Behavior, Social Interaction, or General Locomotor Activity

**Figure 8:**
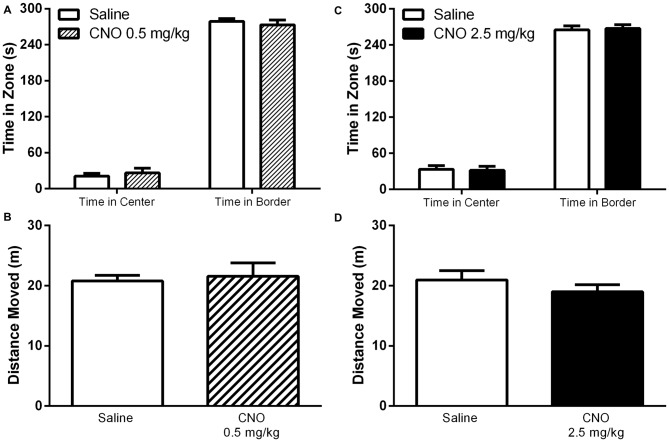
**Exciting mPFC PN does not alter behavior in the open field.** CNO, whether administered at 0.5 mg/kg (striped bars) or 2.5 mg/kg (black bars) had no effect on time spent in the center or time spent in the border of an open field arena, relative to saline (white bars). CNO also had no effect on total distance moved (CNO Panel **A,C**, *N* = 7; Saline Panel **A,C**, *N* = 7; CNO Panel **B,D** *N* = 12, Saline Panel **B,D** *N* = 12). Error bars are ± SEM. Unpaired *t*-test.

**Figure 9:**
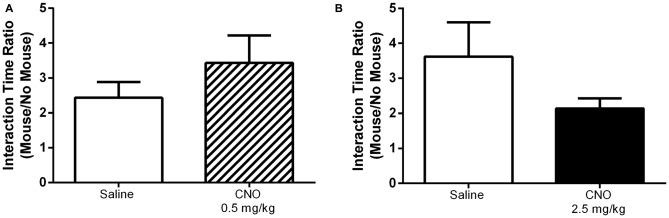
**Exciting mPFC PN does not alter social interaction.** In a social interaction task CNO, whether administered at 0.5 mg/kg (**A**, striped bar, *N* = 8) or 2.5 mg/kg (**B**, black bar, *N* = 10) had no effect on the ratio of time spent interacting with a novel mouse to time spent interacting with an empty animal restrainer, relative to saline (white bars, **(A)** *N* = 8, **(B)** *N* = 12). Error bars are ± SEM. Unpaired *t*-test.

Given that the selective activation of mPFC PN produces effects that are task dependent, we hypothesized that this selectivity might apply to other behaviors previously shown to be modulated by mPFC activity. Indeed, CNO administration had no effect on performance in the open field assay, either at 0.5 mg/kg or 2.5 mg/kg, relative to saline. Specifically, there was no significant effect of CNO on time spent in the center of the arena (Figure 8, 0.5 mg/kg: *P* = 0.5581; 2.5 mg/kg: *P* = 0.8707), time spent in the edges of the arena (Figure 8, 0.5 mg/kg: *P* = 0.5484; 2.5 mg/kg: *P* = 0.8192), or total distance traveled (Figure 8, 0.5 mg/kg: *P* = 0.7566; 2.5 mg/kg: *P* = 0.3292). Excitation of mPFC PN by CNO-DREADD therefore does not alter anxiety or locomotor activity. Furthermore, in a test of social interaction there was no significant difference in exploration of the novel mouse when measured as the ratio of time spent near the restrainer when occupied by novel mouse to time spent near the restrainer when unoccupied (Figure 9). This was true when mice were administered either a low CNO dose (0.5 mg/kg; *P* = 0.2906) or a high CNO dose (2.5 mg/kg; *P* = 0.1994). Our experiments suggest that excitation of mPFC PN by CNO-mediated stimulation of DREADD is not sufficient, by itself, to significantly alter exploratory drive in response to a novel conspecific.

### Targeted Neurons Exhibit Partial Overlap With Drd1-Expressing Population

**Figure 10:**
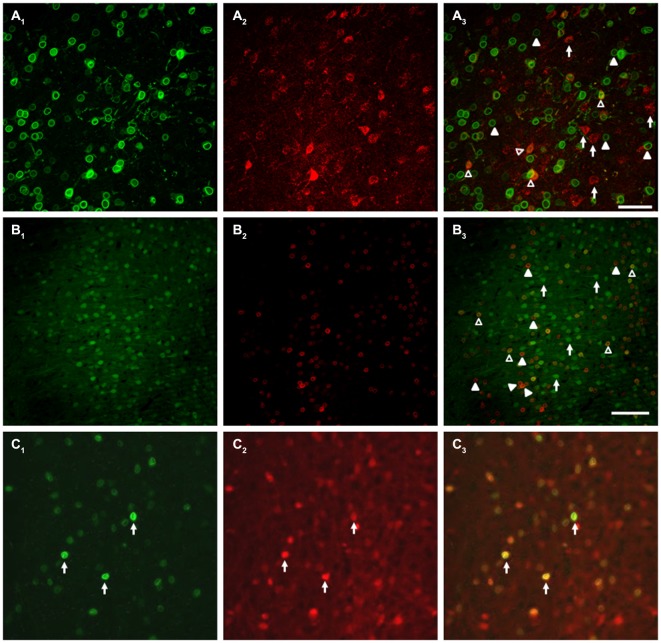
**Targeted neurons exhibit partial overlap with the DR1 expressing population. (A)** Co-injection of AAV-CamKII-hM3D(Gq) and Cre-dependent SUN2-myc into DR1-Cre mice demonstrated that the neurons targeted in this study only partially overlap with the DR1-expressing population. **(A_1_)** SUN2 expression, demonstrating DR1 promoter activity; **(A_2_)** mCherry expression, showing AAV-hM3D(Gq) expression; **(A_3_)** Merged image, with example cells of interest identified. **(B)** A parallel experiment using AAV-CamKII-eYFP instead of AAV-CamKII-hM3D(Gq) showed similar results. **(B_1_)** eYFP epifluoresence; **(B_2_)** SUN2 expression; **(B_3_)** Merged image. In **(A,B)**, Filled arrowheads: DR1/SUN2 expression only; Open arrowheads: dual expression of DR1/SUN2 and eYFP/mCherry/DREADD expression; Arrows: eYFP/mCherry/DREADD expression only. In **(C)**, arrows indicate examples of the expression of both SUN2-myc **(C_1_)**, tdTomato **(C_2_)**, and Merge **(C_3_)** in a control experiment, in VGAT-Cre-tdTomato mice (Vong et al., 2011). No Sun2-myc single positive neurons were observed. Scale bars are 100 μm.

Unconditioned food intake has previously been shown to be sensitive to manipulation of dopamine signaling within the mPFC, particularly through the type 1 dopamine receptor (Drd1, for further details, see “Discussion” Section). Drd1s are expressed on PN in the mPFC, but the extent of this expression is unknown. As we observed no effect of our stimulation on unconditioned food intake, we sought to evaluate whether PN targeted by the DREADD AAV also co-express Drd1s, as a lack of complete overlap could partially explain the absence of unconditioned feeding effects. As existing Drd1 antibodies are unreliable, we developed a Cre-dependent vector in order to be able to label Drd1-expressing neurons in mice expressing Cre recombinase under control of the Drd1 promoter (Drd1-Cre mice). This vector, described in the Methods, encodes a myc-tagged nuclear envelope protein, SUN (Sad1 and Unc domain) 2, which is only functional in cells expressing Cre recombinase. Specificity of the vector was confirmed by a test injection into VGAT-Cre-tdTomato mice (Vong et al., 2011), which demonstrated that SUN2-myc expression was restricted to Cre/tdTomato-expressing cells (Figure 10C). Following this confirmation of vector viability, we coinjected our CamKIIα-driven DREADD AAV, along with SUN2-myc AAV, into the mPFC of DR1-Cre mice. This allowed us to determine the co-expression of both hM3D(Gq) and the Cre dependent nuclear tagged SUN2-myc, which in these experiments served as a proxy for Drd1 expression. As shown in Figure 10A, while many cells expressed both DREADD and SUN2-myc, many cells also expressed only one antigen, indicating that the overlap between DREADD-expressing cells and Drd1-expressing cells is incomplete. These observations were corroborated by a complimentary experiment, involving dual injection of an AAV expressing eYFP under control of the CamKIIα promoter (driving expression in mPFC PN) and an AAV encoding SUN2-myc AAV, which also showed incomplete co-expression (Figure 10B). In order to estimate the extent of overlap, we counted the proportion of DREADD-expressing cells that also expressed SUN2 in a representative sample set, and found that approximately 43.2 ± 6.1% of DREADD-positive cells co-expressed the SUN2-myc marker. This experiment demonstrates that a vector driven by CamKIIα and a vector driven by Drd1-dependent expression of Cre target overlapping, yet distinct neuronal populations within the mPFC.

## Discussion

In our current work we show that increasing the excitability of mPFC PN via DREADD receptor activation is sufficient to enhance performance of behaviors aimed at acquiring highly palatable, rewarding foods. Specifically, we show an increase in operant responding for a palatable food reward, a reduction of premature responding to obtain a food reward, and an increase in reinstatement responding for a highly palatable food. In stark contrast to the behavioral changes observed in these contingent, conditioned reward assays, our PN stimulation paradigm had no effect on food intake under free feeding conditions. To our knowledge, this marks the first demonstration of an mPFC manipulation producing an effect on food-seeking behaviors while simultaneously sparing effects on unconditioned food intake. Furthermore, we observed no effect of PN stimulation on behavior in non-food reward assays: PN stimulation had no effect on locomotion or anxiety behavior. While social interaction is considered to be inherently rewarding for rodents, we observed no effect on behavior in this assay either. These results are therefore consistent with our manipulation producing an increase in the valuation of a food reward, absent effects on free feeding and other measures of behavior. Indeed, the behavioral alterations we have observed following hM3Dq activation (operant conditioning, impulsivity, and reinstatement) all involve relatively high levels of cognitive demand, requiring decision-making, goal directed behavior, and attribution of motivational salience. They all also involve learning and executing a set of behaviors in order to obtain a reward. In contrast, the assays in which we did not observe an effect are relatively non-demanding, and do not require a significant degree of learning or goal/value updating. Thus, increasing PN activation in the medial prefrontal cortex through DREADD signaling, above the level that might be observed during behavioral engagement in less cognitively demanding tasks such as food consumption (Caracheo et al., 2013), does not produce any change in behavioral output. However, during tasks requiring higher cognitive demand (Caracheo et al., 2013), increasing the gain of select PN within the frontal cortex has distinct behavioral consequences. It is worth noting that our treatment improved behavioral performance in a task-specific manner. That is, nosepoking was increased in the operant and reinstatement assay, where nosepoking maximizes reward, whereas nosepoking was decreased in the impulsivity assay, where refraining from nosepoking maximizes reward, indicating that PN stimulation optimized action selection in order to maximize reward receipt.

Interestingly, these data contrast with recent reports suggesting that mPFC neurons do indeed regulate non-contingent food intake. We believe these differences between our observations and those published previously could have resulted for several reasons. First, the cell population targeted in this work, glutamatergic PN, is distinct from those targeted in previous studies that have shown an effect of mPFC manipulation on free feeding. For example, stimulation of μ-opioid receptor-expressing (MOR) neurons in the rat mPFC via infusion of the MOR agonist DAMGO has been shown to increase both free feeding and PR responding for a sucrose reward (Mena et al., 2011; Selleck et al., 2015). In the cortex, MORs are thought to reside predominantly on a subclass of GABAergic interneuron expressing vasoactive intestinal peptide (VIP), a population excluded by our targeting technique (Taki et al., 2000; Férézou et al., 2007; Kubota et al., 2011). Interestingly, PN form the final output pathway of the mPFC, and are therefore downstream of the MOR interneurons. How MOR signaling alters pyramidal neuron activity to alter feeding remains to be determined; however, our stimulation paradigm apparently did not recapitulate the circuit effects of MOR/VIP interneuron stimulation, resulting in an absence of free feeding effects. It is worthwhile noting that stimulation of mPFC MORs with DAMGO actually increases impulsive action in a differential reinforcement of low response rates task, in contrast to our results, which show a decrease in impulsive action upon PN stimulation (Selleck et al., 2015). The partial overlap in behavioral effects of MOR stimulation with pyramidal stimulation, i.e., an increase in operant responding, could be due to a disinhibitory effect of MOR stimulation on pyramidal neuron activity, as is suggested by the circuit structure of mouse visual cortex (Pfeffer et al., 2013); however, in the mPFC this remains to be determined.

In addition to opioid-mediated effects on free feeding, optogenetic stimulation of dopamine type 1 receptor-expressing (Drd1) neurons in the mPFC has also been shown to increase free feeding of both grain pellets and a high fat diet, and stimulation of Drd1 terminals in the amygdala alone is sufficient to increase free feeding on grain pellets (Land et al., 2014). At least some Drd1 neurons in the mPFC co-express MORs (Olianas et al., 2012); whether the two receptors are co-expressed on amgydala-projecting neurons is unknown. In any case, our immunohistochemical study shows that while there is overlap among the populations with active Drd1 and CamKIIα promoters within the mPFC, the majority of neurons in which the CamKIIα promoter can drive AAV-mediated expression (of either eYFP or mCherry) do not co-express a Cre-dependent AAV-delivered payload in Drd1-Cre transgenic mice. Furthermore, our data confirm a divergence in the phenotype of CamKIIα-expressing neurons and the Drd1-expressing population, as not all Drd1-expressing mPFC neurons express a CamKIIα-driven construct. Taken together, we conclude that we are neither targeting all Drd1-expressing neurons in the mPFC, nor does the Drd1-expressing population consist exclusively of PN, and that this may in part explain why targeted stimulation of Drd1-expressing mPFC neurons can enhance free feeding, while targeted stimulation of CamKIIα-expressing mPFC PN does not. As with MORs, the partial overlap between the neurons targeted in this study with those expressing Drd1s may explain commonalities between our observations and those of prior reports. For example, D1R-dependent activity in the mPFC is required for milnacipran-mediated suppression of impulsivity (Tsutsui-Kimura et al., 2013). Our target population includes Drd1-expressing neurons, and so they likely contribute to the suppression of impulsivity that we have observed.

An additional explanation for the divergence in our data from prior reports is the fact that the stimulation technique used in these experiments is physiologically distinct from prior optogenetic experiments that have resulted in alterations in non-contingent feeding. Indeed, our electrophysiological experiments provide valuable new insight into the mechanisms of DREADD signaling while also illustrating the potential role of endogenous muscarinic receptor activation and NO signaling in the modulation of behavior. The hM3D(Gq) DREADD used in this study is derived from the human M3 muscarinic acetylcholine receptor (Armbruster et al., 2007). As predicted, we have found that stimulation of hM3D(Gq) *in vitro* results in neurophysiological responses similar to those induced by M3 stimulation. Notably, CNO dose dependently enhances the generation of action potentials in the prefrontal cortex. As prior work has shown that M3 muscarinic signaling in the mPFC involves the generation of NO (Fassini et al., 2015), we examined whether this mechanism was also important in driving the neuronal activation by CNO. Indeed, we found that the increase in action potential generation was ablated by addition of the NOS inhibitor L-NAME, which further suggests that CNO stimulation may modulate presynaptic vesicle release, as well as producing effects on the postsynaptic neuron. This mechanism of DREADD action likely differs significantly from that observed with the use of optogenetic approaches, as channelrhodopsin acts to directly depolarize the target neuron through an enhancement of sodium and calcium ion influx. Whether optogenetic stimulation, like DREADD, also engages retrograde signaling pathways remains an open question. We speculate that the graded effect of DREADD signaling observed on action potential generation may underlie our observation that operant behavioral responding was dependent upon dosage of the CNO ligand. While a low dose of CNO (0.5 mg/kg) was sufficient to enhance nosepoking for chocolate flavored food pellets, a higher dose (2.5 mg/kg) was required to suppress impulsive responding for a food reward.

Our work also highlights the potential that endogenous muscarinic signaling in the mPFC may produce similar effects on food consumption behavior. Muscarinic receptors are expressed on mPFC PN, and their activation results in neuronal depolarization and activation of NOS (Fassini et al., 2015; Kurowski et al., 2015). Our work demonstrates that driving muscarinic-like receptor activity in the mPFC can modulate reward seeking behavior in the absence of effects on attention. These data are in agreement with the observation that fast nicotinic receptor signaling, rather than metabotropic muscarinic signaling, likely carries information regarding attention and arousal in the mPFC (for review, see Bloem et al., 2014). Further investigation of the role of muscarinic signaling on food intake and food rewarded instrumental behavior is therefore warranted.

Finally, we provide an assessment of the molecular phenotype of 10 DREADD-expressing cells targeted with an AAV vector driven by the CamKIIα promoter. Our results demonstrate that the targeted neurons overwhelmingly display a glutamatergic phenotype, corroborating previous reports using CamKIIα-driven vectors to target glutamatergic PN in this brain region (for example, Van den Oever et al., 2013). Nine out of ten cells expressed either VGLUT1 or VGLUT2, or both (Figure 1C). The presence of these transporters is indicative of an excitatory, glutamatergic phenotype, as VGLUT is required to load synaptic vesicles with glutamate (Münster-Wandowski et al., 2016). Interestingly, we also found that some of these cells expressed mRNA for the markers GAD65, GAD67, PARV, SOM, and vasoactive intestintal peptide (VIP). Although these markers are often ascribed to interneuron populations (for example, Rudy et al., 2011; Xu et al., 2010), our observation of their coexistence with glutamatergic markers in PN is not unprecedented. VGLUT1, VGLUT2, GAD65, and GAD67 have been observed in single cells in a variety of brain regions, across a range of ages (Danik et al., 2005). In fact, PARV-Cre mice show Cre functionality in PN in the frontal cortex, suggesting the presence of PARV in these neurons (Tanahira et al., 2009). Indeed, a subset of corticostriatal projection neurons co-express PARV, VGLUT1, and GAD (Jinno and Kosaka, 2004); this population may account for the PARV-expressing neurons identified in our sample that also co-express both GAD and VGLUT (4/5 cells). Several reports show that PARV, SOM, and VIP are almost always co-expressed with GABAergic markers in frontal cortex, and are only infrequently co-expressed with each other, instead seeming to label distinct populations (Uematsu et al., 2008; Xu et al., 2010). We found that 4/5 PARV-expressing neurons and 4/4 SOM-expressing neurons also expressed GAD, supporting prior observations that PARV and SOM frequently co-occur with GAD. Curiously, we found that 3 of 4 SOM-expressing cells also co-express PARV, in contrast to reports indicating that these markers label distinct populations. While unexpected, this is not unprecedented: a class of neuron expressing VGLUT1, GAD67, PARV, and SOM has previously been characterized in the amgydala (Sosulina et al., 2006). The lone VIP-expressing neuron identified in this study expressed all other markers tested except VGLUT2. Some mPFC neurons that project to the nucleus accumbens are known to express VIP, but whether they also contain PARV and SOM is unknown (Lee et al., 2014). While 9/10 cells expressed VGLUT, a single GAD+/PARV+ cell did not (Cell 6). Despite the apparent lack of a glutamatergic phenotype in this cell, it may indeed belong to a recently identified population of VGLUT−/GAD+/PARV+ cells that project from the mPFC to the nucleus accumbens (Lee et al., 2014). In summary, the scRT-PCR data indicate that we have predominantly, if not exclusively, targeted pyramidal projection neurons. Overall, our observations indicate significant molecular diversity among the pyramidal cell population of the mPFC. The co-expression of VGLUT and GAD in mPFC PN is likely important for proper execution of mPFC function (Münster-Wandowski et al., 2016). It is possible that the diverse pyramidal subtypes subserve different components of the behaviors we have assessed. The phenotypic distinctions among mPFC PN present opportunities for future intersectional studies of pyramidal neuron subtypes in controlling feeding behavior and other aspects of PFC function.

In conclusion, our work demonstrates for the first time that DREADD-driven neuronal stimulation is sufficient to enhance both the effort devoted to food seeking and the reinstatement of cued food seeking behavior, while reducing motor impulsivity. Surprisingly, no change in affect or food intake was observed, suggesting that activation of CamKII PN using a Gq coupled DREADD drives food seeking behavior but not the ingestion of food.

## Author Contributions

DMW: wrote the manuscript, designed and performed experiments. MO, YS, BSB, PSL, RPG, BAN, EP-R: performed experiments described in the manuscript. YO: designed programs used to measure impulsivity and attention. ADG: designed experiments described in the manuscript and contributed to the writing of the manuscript. MKP: performed experiments described in the manuscript and participated in the writing of the manuscript. MMS: designed the experiments described in the manuscript and participated in the writing of the manuscript. All authors listed, have made substantial, direct and intellectual contribution to the work, and approved it for publication.

## Conflict of Interest Statement

The authors declare that the research was conducted in the absence of any commercial or financial relationships that could be construed as a potential conflict of interest.
